# Dermal fibroblasts display similar phenotypic and differentiation capacity to fat-derived mesenchymal stem cells, but differ in anti-inflammatory and angiogenic potential

**DOI:** 10.1186/2045-824X-3-5

**Published:** 2011-02-08

**Authors:** Antonella Blasi, Carmela Martino, Luigi Balducci, Marilisa Saldarelli, Antonio Soleti, Stefania E Navone, Laura Canzi, Silvia Cristini, Gloria Invernici, Eugenio A Parati, Giulio Alessandri

**Affiliations:** 1 Medestea Research and Production Laboratories, Consorzio Carso, 70010 Valenzano, Bari, Italy; 2 Cellular Neurobiology Laboratory, Department of Cerebrovascular Diseases, Fondazione IRCCS Neurological Institute "Carlo Besta", 20133 Milan, Italy

## Abstract

**Background:**

Mesenchymal stem cells (MSCs) are multipotent stem cells able to differentiate into different cell lineages. However, MSCs represent a subpopulation of a more complex cell composition of stroma cells contained in mesenchymal tissue. Due to a lack of specific markers, it is difficult to distinguish MSCs from other more mature stromal cells such as fibroblasts, which, conversely, are abundant in mesenchymal tissue. In order to find more distinguishing features between MSCs and fibroblasts, we studied the phenotypic and functional features of human adipose-derived MSCs (AD-MSCs) side by side with normal human dermal fibroblasts (HNDFs) *in vitro*

**Methods:**

AD-MSCs and HNDFs were cultured, expanded and phenotypically characterized by flow cytometry (FC). Immunofluorescence was used to investigate cell differentiation. ELISA assay was used to quantify angiogenic factors and chemokines release. Cultures of endothelial cells (ECs) and a monocyte cell line, U937, were used to test angiogenic and anti-inflammatory properties.

**Results:**

Cultured AD-MSCs and HNDFs display similar morphological appearance, growth rate, and phenotypic profile. They both expressed typical mesenchymal markers-CD90, CD29, CD44, CD105 and to a minor extent, the adhesion molecules CD54, CD56, CD106 and CD166. They were negative for the stem cell markers CD34, CD146, CD133, CD117. Only aldehyde dehydrogenase (ALDH) was expressed. Neither AD-MSCs nor HNDFs differed in their multi-lineage differentiation capacity; they both differentiated into osteoblast, adipocyte, and also into cardiomyocyte-like cells. In contrast, AD-MSCs, but not HNDFs, displayed strong angiogenic and anti-inflammatory activity. AD-MSCs released significant amounts of VEGF, HGF and Angiopoietins and their conditioned medium (CM) stimulated ECs proliferation and tube formations. In addition, CM-derived AD-MSCs (AD-MSCs-CM) inhibited adhesion molecules expression on U937 and release of RANTES and MCP-1. Finally, after priming with TNFα, AD-MSCs enhanced their anti-inflammatory potential; while HNDFs acquired pro-inflammatory activity.

**Conclusions:**

AD-MSCs cannot be distinguished from HNDFs *in vitro *by evaluating their phenotypic profile or differentiation potential, but only through the analysis of their anti-inflammatory and angiogenic properties. These results underline the importance of evaluating the angiogenic and anti-inflammatory features of MSCs preparation. Their priming with inflammatory cytokines prior to transplantation may improve their efficacy in cell-based therapies for tissue regeneration.

## Background

Within the panorama of adult stem cells, mesenchymal stem cells (MSCs) have been proposed for application in cell-based therapies because of their multipotential differentiation capacity, easy tissue accessibility and capacity for ex-vivo expansion [1]. MSCs are localized in the stroma of tissue or organs, and they have been isolated and characterized from different adult tissue sources such as bone marrow (BM), skeletal muscle, pancreas, vessels, dental pulp and adipose tissue [2-7]. Different methods for their isolation and culture expansion have been described [3,4,8]. The majority of techniques used direct adhesion to tissue culture plastic to separate MSCs from the unwanted cells contained in the stroma [5,8]. This procedure can be easily applied to separate MSCs from BM or umbilical cord blood. In fact, unwanted cells continue to float in the culture medium and can be easily discarded by replacing the culture medium. The technique to separate MSCs from not-hematopoietic tissue, for example adipose tissue, is more complex because MSCs are only a small cell subpopulation of the total stromal cells that adhere to plastic after seeding. In particular, fibroblasts are adherent proliferating cells which are difficult to remove because they can survive and grow even under extremely selective culture conditions.

Fibroblasts are considered mature mesenchymal cells that are particularly abundant in the connective of each organ and tissue. Therefore, these cells are the most frequent contaminating cell phenotype present in many cell culture systems [9]. Not only is it difficult to apply techniques which successfully eliminate fibroblasts from a culture, it is also particularly complex to distinguish MSCs from fibroblasts in the same culture. Fibroblasts and MSCs have an extremely similar morphological appearance [10]; they both proliferate well and have many identical cell surface markers [10]. MSCs lack a specific surface antigen that precisely differentiates these cells from fibroblasts. Stro-1, and more recently CD146, have been claimed as specific markers for MSCs [11-13 ]. However, these markers seem limited to MSCs derived from BM (BM-MSCs) or from renal tissue, since adipose-derived MSCs (AD-MSCs), for example, did not display these markers [14,15]. In spite of these differences, MSCs of different tissue origin possess a very similar phenotypic profile. They usually express high levels of mesenchymal markers and in general do not express hematopoietic markers [14,15]. Until now, the best way to distinguish MSCs from fibroblasts is based on the analysis of the functional properties of these two kinds of cells; MSCs self-renew and retain multipotent differentiation capacity, while fibroblasts seem more limited in both these functional properties [14]. However, a deeper comparative analysis between MSCs and fibroblasts is lacking. In particular, given the importance of the application of AD-MSCs in cell based therapies, it is relevant to distinguish them from fibroblasts which significantly contribute to the stromal cell population of adipose tissue. In order to fill this gap and to find functional features that may better help to distinguish culture of AD-MSCs from fibroblasts, in the present work we investigated the phenotypic and functional properties of three different preparations of human AD-MSCs and two normal human dermal fibroblast (HNDFs) cell lines side by side. We confirm that HNDFs and AD-MSCs display similar phenotypic and functional features, including the capacity to differentiate into different cell lineages. We here demonstrate for the first time that, while AD-MSCs possess a strong anti-inflammatory and angiogenic potential, HNDFs do not.

## Methods

### Isolation and culture of human AD-MSCs and Fibroblasts

Human fat specimens were excised from 3 patients after approval by the ethics committee of "MIULLI" General Regional Hospital (Acquaviva, Bari). Informed consent was obtained from each human donor. Adipose tissue samples were collected under sterile conditions from 3 male donors (aged from 40 and 60 years) who underwent standard laparoscopic surgery (uncomplicated colecistectomy and inguinal hernia), after a negative blood screening for HIV, HBV, HCV, Treponema pallidum, HTLV-I and II. Each adipose tissue sample was treated and cultured separately.

Human AD-MSCs were isolated as described [3,15]. Briefly, sterile adipose tissue samples were transferred in 50 ml falcon tubes containing PBS plus penicillin-streptomicine solution and quickly transferred at 4°C temperature to our laboratories. Each sample was repeatedly washed with PBS for blood residual and connective tissue removal. Subsequently, fat specimens were minced with scissors and washed with PBS by centrifugation at 250 × g. After liquid phase removal, collagenase solution (0,25% w/v) (SIGMA St. Louis, Mo, USA) plus 200 μl DNAase (SIGMA) at 1:100 dilution were added to pellets. All tubes were then incubated at 37°C for 2 hours. After enzymatic digestion, cells were washed by centrifugation at 250 × g for 10'. The pellets were resuspended in DMEM +10% FBS medium, filtered (100 μm Ø) to remove undigested tissue, seeded into T 25 culture flasks and incubated at 37°C in a humidified atmosphere containing 5% CO2. The following day, the medium was aspirated and the adherent AD-MSCs were cultured in EGM medium supplemented with 10% FCS, 50 ng/ml bFGF and antibiotics (Lonza, Verviers, Belgium). The two normal human adult dermal fibroblasts (HNDFs) cell lines were purchased from Lonza (Verviers, Belgium catalogue N° CC25) and cultured under identical conditions used for AD-MSCs. Cultures were routinely passed at 70-80% of confluence and, for this study, cultures were not expanded for more than 8-10 *in vitro *passages. The growth rate of each AD-MSCs preparation and for HNDFs was determined as previously described [15]. Briefly, AD-MSCs and HNDFs (at passage 4) were harvested from the culture flask by treatment with trypsin (Sigma) in PBS (0.05% w/v). Following enzyme inactivation and centrifugation, cells were resuspended in EGM +10% FCS and 50 ng/ml bFGF and seeded at a final density of 2 × 10^4 ^cells/ml in T25 culture flasks. After a 7-day incubation, AD-MSCs and HNDFs were detached and counted by hemocytometer.

### Characterization of humanAD-MSCs and HNDFs

Cultures of AD-MSCs and HNDFs were characterized by flow cytometry (FC). After trypsinization, cells were resuspended with FC buffer (pH 7.2 PBS, BSA 0.5%, Sodium Azide 0.02%) and fixed with paraformaldehyde 2%. AD-MSCs and HNDFs were marked at concentration of 1 × 10^5^/500 μl. For phenotypic analysis, fluorescein isothiocyanate (FITC-F), phycoerythrin (PE) or phycoerythrin-cyanin PC5 conjugate-antibodies were used. The expression of the following markers were investigated: CD44F, CD90PE, CD34PE, CD45F, CD54F, CD146PE, CD117PC5, CD31F, vWf-F, CD105PE, CD106PE, CD166F, CD40F, CD80F, CD86PE, FAS (CD95 member of the family of TNFα's receptors), FAS-L (CD178 ligand of FAS) (Immunotech^®^, Milan, Italy), CD56PE (Serotec^®^, Italy), HLAIF, HLAIIPE, CD29PE, CD49dPE (Biolegend^®^, Italy), Stro-1PE (R&D^®^, Milan, Italy), CD133 (Miltenyi Biotec^®^, Bologna, Italy). AD-MSCs and HNDFs were incubated for 20'/room temperature in a dark room and then cells were washed with FC buffer to remove not-conjugated antibodies. Epics "XL-MCL" (Beckman Coulter USA) flow cytometer was used for simultaneous forward (FSC) and side scatter (SSC) measuring, and analyzing the multiparametric fluorescent phenotypic marker signals. 20,000 events were acquired for each analysis. Sample histogram elaboration was performed with EXPO 32 software to assess fluorescent distribution.

Aldefluor test (Aldagen, Inc., North Carolina, USA) was used to identify, evaluate, and isolate cells with low side scatter which expressed high levels of aldehyde dehydrogenase (ALDH). ALDH cells have been shown to have properties of stem and progenitor cells [16].

The cellular samples were adjusted to a concentration of 1 × 10^6 ^cells/ml with Assay Buffer provided in the kit. The adjusted cell suspension was placed into each "test" sample tube.

DEAB (diethylaminobenzaldehyde) solution, provided in the kit, was added to the "control" tube. The samples were incubated for 40' at 37°C in the dark. Following incubation, the tubes were centrifuged for 5' at 250 × g. The pellets were suspended in 0.5 ml of Aldefluor Assay Buffer provided by the kit and analyzed immediately by FC.

### Differentiation of AD-MSCs and NHDFs

To examine the capacity of AD-MSCs and HNDFs to differentiate toward adipogenic, osteogenic and cardiomyogenic cell lineages, lineage-specific induction factors were used. For osteogenic differentiation, AD-MSCs and HNDFs were cultured in the presence of DMEM/F-12 supplemented with 10% FBS, 0.1 mM dexamethasone (Sigma), 10 mM β-glycerolphosphate (Sigma) and 50 mM ascorbic acid (Sigma) for around 2 weeks. At the end of incubation, osteogenic differentiation was assessed by alkaline phosphatase (AP) expression (Miltenyi Biotec^®^). Fully differentiated osteoblast-like cells generated from AD-MSCs and *HNDFs *changed their original fibroblastic shape into a cubical/epithelial shape with prominent cytoplasmic extensions similar to an osteblast morphology. Cells stained for AP activity with NBT substrate appeared purple in color. Adipogenic differentiation was obtained upon cultivation of AD-MSCs and HNDFs in DMEM supplemented with 10% FBS, 1 mM dexamethasone (Sigma), 0.5 mM methyl-isobutylxanthine (Sigma), 10 mg/ml insulin (Invitrogen, Carlsbad, CA) and 100 mM indomethacin (Sigma) for 3 weeks. At the end of incubation, adipogenic-like cells were revealed by staining with Oil Red O (Sigma). Moreover, to confirm adipogenic differentiation, we analyzed the production of Adiponectin by HNDFs and AD-MSCs by FC using anti-Adiponectin antibodies (Sigma). Cardiomyogenic differentiation of AD-MSCs and HNDFs was assessed by immunofluorescent assay by testing the expression of anti-Desmin (Signa), anti-Troponin C-cardiac (TNP-C), anti-Myocardial actin (MyoA), anti-Connexin 43 (Cx43) and anti-Atrial Natriuretic factor, all purchased from Biodesign International (Saco, ME, USA). Cardiomyogenic markers expression on AD-MSCs and HNDFs was analyzed upon 3 weeks of culture in EGM complete medium in the presence or in the absence of 1 μ/ml 5-azacytidine (Sigma) as previously described [15]. Briefly, after washing, the cells were incubated with secondary antibodies Beckman Coulter FITC-conjugated Goat anti Mouse-IgG (Immunotech^®^, Milan, Italy) for 2 hours at room temperature. Then the cells were washed to remove excess antibodies. In order to stain the nuclei, samples were incubated with DAPI (dilution 1:10,000 in PBS, Sigma) for 8' at room temperature. As negative controls, irrelevant isotype-matched antibodies were used. The slides were then mounted in Gel Mount (Biomedia, Foster City, CA) and sealed. Fluorescence analysis was established with fluorescence inverted microscope Leica DM IL (Leica Microsystems GmbH, Wetzlar, Germany). The images were adjusted in contrast and brightness with the image processing module "Ambient LAS" for the acquisition and overlay of fluorescent images (Leica Microsystems GmbH, Wetzlar, Germany).

### Evaluation of Growth factors and cytokines production by AD-MSCs and HNDFs

ELISA-tests were performed to detect growth factors and cytokines released by AD-MSCs and HNDFs in the culture medium. Aliquots (2 ml) of conditioned medium (CM) from AD-MSCs (AD-MSCs-CM) and HNDFs (HNDFs-CM) cultures were collected after 72 hours at 70-80% of cell confluence and between 5-8 in vitro passages. Afterwards, AD-MSCs and HNDFs were detached with trypsin, counted and stained with Trypan Blue to detect cell viability and to normalize the amount of released factors in respect to the number of cells.

The growth factors, Vascular Endothelial Growth Factor (VEGFa), Transforming Growth Factor β1 (TGF-β1), Hepatocyte Growth Factor (HGF), Platelet-derived Growth Factor (PDGF), Angiopoietin 1 (Ang-1) and 2 (Ang-2), IL6 and IL8 production were quantified with ELISA-kits (R&D Systems, UK, Europe), according to the manufacturer's instructions.

The specific protein concentration in CMs was detected in accordance with the standard guideline protocol supplied with ELISA kit. The background value of each growth factor analyzed and contained in the control medium was subtracted. Absorbance was measured at 450 nm with a microplates photometric reader DV990BV4 (GDV, Italy).

Data were expressed as mean ± SD of the secreted factor per 10^6 ^cells. Assay was repeated twice and each sample was run in triplicate.

### Evaluation of Angiogenic potential of AD-MSCs and HNDFs

To assess the angiogenic potential of AD-MSCs and HNDFs, we investigated the capacity of their CMs on the proliferation of Human Umbilical Vein ECs (HUVECs) (Lonza) and Human derma-derived microvascular ECs (HMECs) isolated as previously described [17]. Both ECs phenotypes were routinely maintained in EGM bullet kit (Lonza) plus 10% FCS. ECs proliferation assay was performed as described [17]. Briefly, HUVECs and HMECs (at passage 3) were harvested from culture flasks by trypsin. After enzyme inactivation and centrifugation, cells were resuspended in EGM medium +0.2% BSA and counted. To evaluate the growth response to AD-MSCs-CM and HNDFs-CM, 0.5 ml of HUVECs and HMECs (10^4 ^cells) were seeded into each well of a 24-multiwell plate coated with collagen type I; after cell adhesion, medium was aspirated and replaced with EGM complete medium (+10% FCS + 50 ng/ml bFGF) supplemented or not with different dilutions of CM. Positive control growth medium consisted in EGM complete medium plus 10 ng/ml of VEGFa (Lonza). After 72 h, the wells were washed and the cells were fixed and stained. The cells were counted with a calibrated ocular eyepiece in 10 different fields at 400× magnification. Every test was run in triplicate, and at least 3 different CM preparations were tested for both AD-MSCs and HNDFs.

To test the effect of AD-MSCs-CM and HNDFs-CM on ECs tube formation, we used growth factor reduced-matrigel assay (Sigma) as described by Kleinman et al [18]. Briefly, around 50 ul of matrigel were seeded into cold (maintained at 4°C) wells of a 96-multiwell plate. After matrigel jellification at 37°C for 30', HUVECs and HMECs were seeded on matrigel at concentration of 10^4 ^cells/well in 50 ul of EGM control growth medium diluted (1:1) or not with AD-MSCs-CM and HNDFs-CM. On day 2 and 5 after seeding, the number of EC tube formations were counted at 10× magnification by inverted microscopy and reported as n° of tube structures/field.

### Evaluation of anti-inflammatory activity of AD-MSCs and NHDFs

The anti-inflammatory activity of AD-MSCs and HNDFs was tested by applying AD-MSCs-CM and HNDFs-CM on the U937 monocyte cell line (ATCC Manassas VA, USA), both in the absence and the presence of inflammatory stimuli LPS 1 μg/ml (Sigma) and TNFα 25 ng/ml (Sigma). The expression of the adhesion molecules CD54, CD44, CD62L and CD49d on U937 were analyzed by FC. Briefly, U937 cells cultured in RPMI (Sigma) +10% FCS were counted and diluted to a concentration of 2 × 10^5^/ml. In a first series of experiments, U937 were cultured in the presence or absence of different dilutions of AD-MSCs-CM and HNDFs-CM for 24 hours. Afterwards, the cells were washed and pulsed with TNFα 25 ng/ml for a further 12 hours. At the end of incubations, U937 were treated, for 20'/room temperature in dark room, with the different adhesion molecules antibodies. After washing cells to remove not-conjugated antibodies, adhesion molecules expression was analyzed by FC. Using a similar procedure, we also evaluated the adhesion molecules expression on AD-MSCs and on HNDFs pulsed or not for 12 hours with TNFα 25 ng/ml. ELISA-kits were used to quantify the production of RANTES and MCP-1 (R&D Systems, UK, Europe) by U937 under basal culture conditions, in the presence of inflammatory stimuli (LPS 1 ug/ml and TNFα 25 ng/ml) and in the presence of AD-MSCs-CM and HNDFs-CM. Finally, we also evaluated the effect of AD-MSCs-CM and HNDFs-CM on RANTES and MCP-1 release by U937, after priming with TNFα. Briefly, AD-MSCs and HNDFs were exposed for 12 hours to TNFα 25 ng/ml. Thereafter, cells were washed and further incubated for 24 hours. At the end of incubation, their CMs were collected and tested on U937 chemokines production. All the data were normalized for 10^6 ^cells in 24 hours incubation. and the basal level production of the same chemokines present in the AD-MSCs-CM and in HNDFs-CM was subtracted.

### Statistic analysis

Results are expressed as mean ± standard deviation (SD). Statistical significance was evaluated by analysis of variance followed by Tukey-Kramer multiple comparison test and by Student's t-test. A p value of less than 0.05 denotes statistical significance.

## Results

### Culture of AD-MSCs and HNDFs showed a very similar morphological appearance and phenotypic profile

AD-MSCs were isolated from subcutaneous fat specimens obtained from 3 patients during the course of abdominal surgery. AD-MSCs were cultured using EGM medium supplemented with 10% FCS and bFGF. Successful isolation and culture expansion of AD-MSCs were obtained from all three specimens processed. HNDF cell lines were maintained *in vitro *under the same culture conditions of AD-MSCs. HNDFs and AD-MSCs proliferated well in culture with an average doubling time of 28-36 hrs and 38-46 hrs respectively. Cells were usually plated in T25 culture flasks at a density of 20,000 cells/cm^2^. Soon after culture, AD-MSCs showed typical fibroblast-like morphology and did not display any particular morphological differences when compared to HNDFs, even upon 10 *in vitro *passages (Additional file 1: Figure S1).

The phenotypic profile of AD-MSCs and HNDFs was determined by FC analysis and was performed upon 5 *in vitro *passages. At first, we analyzed the presence of stem cell markers such as CD34, Stro-1, CD146, CD117, CD133 and ALDH. As reported in Table 1, neither AD-MSCs nor HNDFs expressed the stem cell markers investigated, only ALDH was significantly expressed in approximately 40% of the cells, but again, there was no substantial difference between AD-MSCs and HNDFs (Additional file 2: Figure S2)

**Table 1:** Phenotypic characterization of human AD-MSCs and HNDFs

	AD-MSCs	HNDFs
	% of positive cells (±SD)	% of positive cells (± SD)
*Stem cell markers*		
Stro-1	3.7 ± 3.2	5.5 ± 0.4
CD146	1.5 ± 1.0	1.2 ± 1.2
CD133	0.4 ± 0.3	absent
CD34	absent	absent
CD117	0.1 ± 0.1	absent
ALDH	42 ± 8.3	41.0 ± 5.4
		
*Mesenchymal markers*		
CD105	98.4 ± 1.0	95.6 ± 2.2
CD90	97.6 ± 2.5	94.1 ± 3.2
CD44	92.5 ±3.6	97.1 ± 1.1
CD29	95.5 ± 2.0	91.1 ± 5-4
		
*AM markers*		
CD166	66.1 ± 26.4	80.4 ± 5.8
CD106	25.2 ± 15.1	35.6 ± 7.8
CD54	19.6 ± 9.4	25.7 ± 15.5
CD56	17.8 ± 12.2	39.6 ± 5.6*
CD49d	10.8 ± 3.4	58.9 ± 22.4**
		
*Endothelial markers*		
CD31	absent	absent
vWf	absent	absent
		
*Immunological markers*		
CD40	17,7 ± 5,9	84.1 ± 3.6**
CD80	4.6 ± 1.1	2.1 ± 0.9
CD86	2.6 ± 1.1	0.8 ± 0.7
CD45	absent	absent
HLA-II	0.5 ± 0.5	1.1 ± 1.2
HLA-I	93.3 ± 3.3	94.0 ± 2.8

Table shows the phenotypic characterization of AD-MSCs and HNDFs in culture. FACS analysis was performed when all cultures were at 5 in vitro passages. Data represent the % of positive cells for each marker analyzed on AD-MSCs and on HNDF and are means ± SD. Variability of each marker tested among the different cell preparations were ≤ 10%. Note that some markers such as CD56, CD49d and CD40 are more highly expressed in HNDFs when compared to AD-MSCs. *p < 0.05 and **p < 0.01 compared to AD-MSCs.

In Table 1 all the results on the phenotypic characterization of HNDFs and AD-MSCs regarding mesenchymal, endothelial and immunogenic markers (see also Additional file 3: Figure S3) are summarized. The variation of each marker among the 3 different AD-MSC preparations and the two HNDF cell lines analyzed was less then 10%. Cells were almost 100% positive for CD29, CD90, CD44 and CD105 mesenchymal markers, and were practically negative for EC markers CD31 and vWf. AD-MSCs and HNDFs had a very similar adhesion molecules profile, showing a variable positivity for markers such as CD166, CD54, CD106 and CD56. Interestingly, only CD49d, an integrin involved in the homing of cells to an inflammatory site [19], was significantly more highly expressed on HNDFs compare to AD-MSCs. Finally, the comparative analysis of markers involved in immune response demonstrated again a strong similarity between AD-MSCs and HNDFs. They both expressed very low levels of CD80 and CD86 and they lacked CD45 and HLA-II expression, while HLA-I was present in 100% of the cells. Interestingly CD40, a co-stimulatory protein found on antigen presenting cells (APCs) [20], was more highly expressed on HNDFs, suggesting a higher immunogenic profile of HNDFs if compared with AD-MSCs. This data further confirms previous results showing the positive expression of CD40 on dermal fibroblasts [21]

### AD-MSCs and HNDFs display similar differentiation capacity

**Figure 1:**
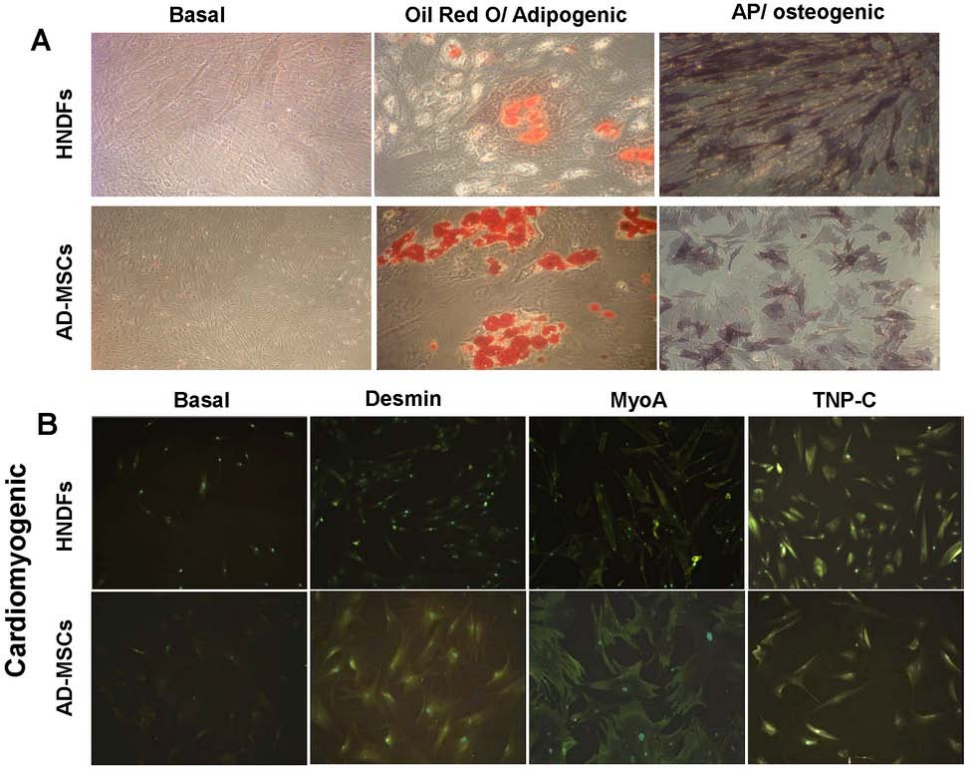
**AD-MSCs and HNDFs display similar capacity to differentiate into adipogenic, osteogenic and cardiomyogenic cell lineages**. The Figure shows the capacity of AD-MSCs and HNDFs to differentiate toward the adipogenic, osteogenic and cardiomyogenic cell lineages in the presence of lineage-specific induction factors. In **(A) **is shown the positive staining of AD-MSCs and HNDFs for Oil Red O and AP, indicating differentiation into adipogenic and osteogenic cell lineages respectively (magnification 20×). In **(B) **note the positive immunofluorescence staining of AD-MSCs and HNDFs for Desmin, MyoA and TNP-C cardiomyogenic markers (magnification 10×).

A typical feature of MSCs is their mutipotential differentiation capacity [2,3]. Previous reports indicate that MSCs of different tissue origin have the capacity to differentiate into different cell lineages, while the multipotential differentiation capacity of fibroblasts is more controversial [14]. Therefore, we compared the capacity of AD-MSCs and HNDFs to differentiate into different cell lineages. Under the same differentiation culture conditions, AD-MSCs and HNDFs were induced to differentiate into osteogenic, adipogenic and cardiomyocyte-like cells. As shown in Figure 1, AD-MSCs and HNDFs differentiate well into osteoblast-like and into adipocyte-like cells assessed by the positivity to AP and Oil Red O staining respectively (Figure 1A). To further confirm adipogenic differentiation, AD-MSCs and HNDFs were also analyzed for Adiponectin expression, a hormone specifically produced by mature adipocyte [22]. Upon adipogenic differentiation, we found a similar significant increment of Adiponectin expression in both AD-MSCs and HNDFs (Additional file 4: Figure S4). They both differentiated into cardiomyocyte-like cells, although a more intense staining of MyoA, TNP-C and Desmin on AD-MSCs was observed (Figure 1B). Other myocardial markers, such as Cx43 and atrial natriuretic factor, were not detected on either HNDFs or AD-MSCs (data not shown).

### AD-MSCs, but not HNDFs, possess strong angiogenic potential in vitro

**Figure 2:**
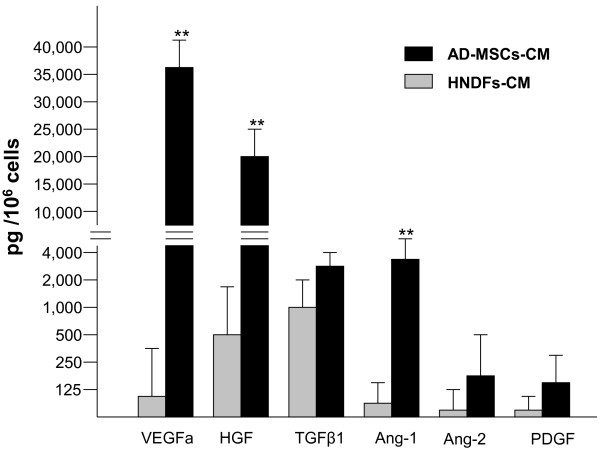
**AD-MSCs, but not HNDFs, produce high levels of angiogenic factors**. ELISA-tests were performed to detect VEGFa, TGF-β1, HGF, PDGF, Ang-1 and Ang-2 angiogenic factors released by AD-MSCs and HNDFs in the CM. Note that AD-MSCs, compared to HNDFs, release very high quantities of VEGFa, HGF and Ang-1. Data are the absolute values expressed as mean ± SD of the secreted factor per 10^6 ^cells after 72 hrs of incubation. The background values of angiogenic factors contained in EGM control medium (supplemented with 10% FCS and 50 ng/ml bFGF) were subtracted. Tests were run in triplicate and repeated twice. ** p < 0.01 *versus *HNDFs release

To define the angiogenic activity of AD-MSCs and HNDFs, we first analyzed the production of angiogenic growth factors and cytokines in their CM. AD-MSCs and HNDFs were cultured for 72 hours, CM was then analyzed by ELISA for the presence of VEGFa, HGF, TGFβ1, Ang-1, Ang-2 and PDGF. AD-MSCs demonstrated a great capacity to produce a broad spectrum of angiogenic factors (Figure 2). In 72 hours of culture, 10^6 ^cells produced an extremely high level of the angiogenic factors HGF and VEGFa. AD-MSCs produced also significant amounts of TGFβ1, Ang-1 and Ang-2 and, to a minor extent, PDGF. Interestingly, HNDFs had a significantly lower production of all the angiogenic factors analyzed. Compared to AD-MSCs, VEGF, HGF and Ang-1 production in HNDFs was significantly lower. We also analyzed the production of angiogenic cytokines, such as IL6 and IL8, which were produced in good quantities by both kinds of mesenchymal cells (Additional file 5: Figure S5).

**Figure 3:**
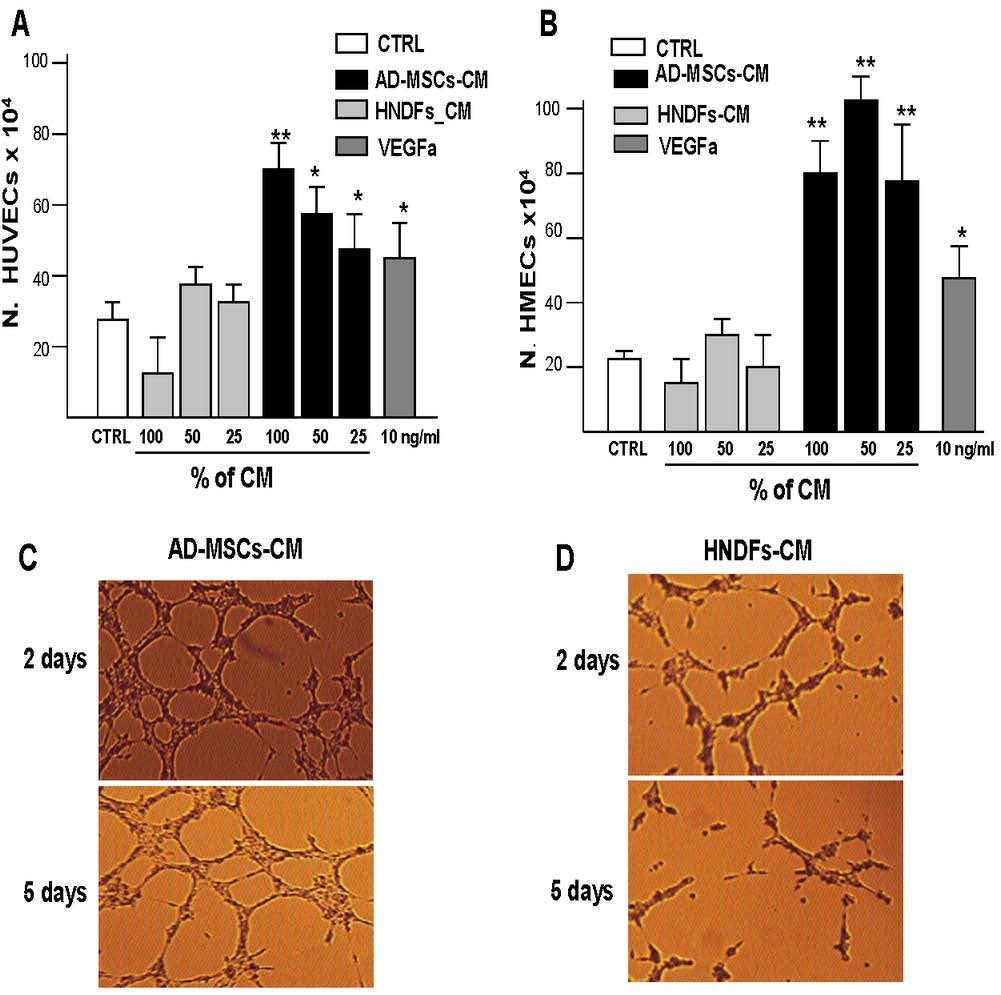
**AD-MSC-CM possess a higher capacity to stimulate HUVECs and HMECs proliferation and tube formations compare to HNDFs-CM**. The effect of AD-MSCs-CM and HNDFs-CM on proliferation of HUVECs **(A) **and HMECs **(B) **was tested by adding different dilutions to EGM control medium. As a positive growth control, we used VEGFa 10 ng/ml. AD-MSCs-CM addition had a great capacity to stimulate the proliferation of HMECs **(B) **and to a minor extent HUVECs (**A**), while HNDFs-CM addition did not affect ECs proliferation at any dilution tested. In **(C) **and in **(D) **are shown the capacity of AD-MSCs-CM and HNDFs-CM to induce HMECs tube formation respectively. AD-MSCs-CM and HNDFs-CM were added at a ratio 1:1 with EGM control medium (Magnification 10×). Note that AD-MSCs-CM not only increased HMECs tube formation on day 2, but also delayed their regression on day 5. The columns in (**A) **and (**B) **are mean ± SD of three independent experiments run in triplicate. *p < 0.05 and **p < 0.01 versus CTRL.

The angiogenic capacity of AD-MSCs and HNDFs was also evaluated on the proliferation of HUVECs and HMECs. As shown in Figure 3, the addition of AD-MSCs-CM, at all different dilutions tested, enhanced growth of both HUVECs (Figure 3A) and HMECs (Figure 3B). In contrast, the addition of different dilutions of HNDFs-CM did not enhance the proliferation of either HUVECs (Figure 3A) or HMECs (Figure 3B). The capacity AD-MSCs and HNDFs to stimulate angiogenesis was also tested *in vitro *by evaluating the formation of capillary-like structures on matrigel. AD-MSCs-CM and HNDFs-CM were added to HMECs cultured on matrigel, a matrix that induces capillary-like structure formation that usually regresses within 3-4 days of culture [18]. As shown in Figure 3C and 3D, within 2 days, the presence of AD-MSCs-CM in the control medium substantially triplicated (AD-MSCs-CM 25 ± 17 versus HNDFs-CM 8 ± 6) the number of HMECs tube formations. Moreover, compared to HNDFs-CM, AD-MSCs-CM produced a significant delay of capillary-like structures regression (Figure 3C and 3D). The effect of AD-MSCs-CM on HUVECs was similar (data not shown).

### AD-MSCs, but not HNDFs, possess strong anti-inflammatory activity in vitro

**Figure 4:**
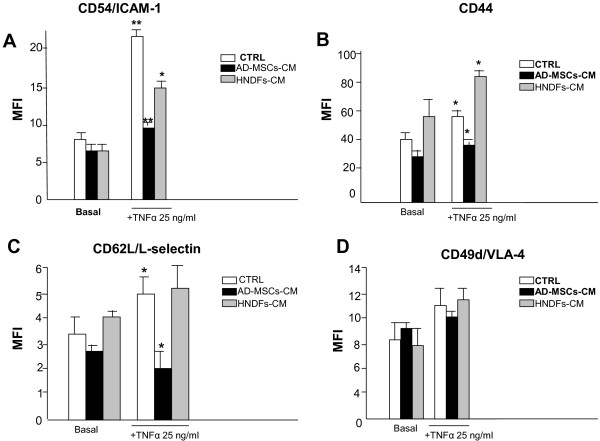
**AD-MSCs, but not HNDFs, reduced expression of AMs on U937**. AD-MSCs-CM and HNDFs-CM were tested on the adhesion molecules expression of the U937 monocyte cell line stimulated or not with TNFα (25 ng/ml × 12 hrs). Mean Fluorescent Intensity (MFI) of each marker expression was evaluated by FACS and values are the mean ± SD of independent experiments performed with 3 different preparations of AD-MSCs-CM and HNDFs-CM. In **(A) **the expression of CD54/ICAM-1, **(B) **the expression of CD44, **(C) **the expression of CD42L/L-selectin and in **(D) **the expression of CD49d/VLA-4. Note that, both AD-MSCs-CM and HNDFs-CM did not affect basal adhesion molecules expression on U937. However, AD-MSCs-CM, but not HNDFs-CM (at 1:1), inhibited CD54/ICAM-1, CD44 and CD42L/L-selectin up-modulation produced by TNFα stimuli. CD49d expression was not affected by either AD-MSCs-CM or HNDFs-CM. * p < 0.05 and **p < 0.01 versus CTRL.

It has been shown that MSCs display anti-inflammatory capacity [15,23,24]. We thus investigated whether AD-MSCs and HNDFs may produce molecules with anti-inflammatory activity by testing their CM on U937, a human monocyte cell line, under basal culture conditions or in the presence of TNFα inflammatory stimuli. As shown in Figure 4, the expression of the adhesion molecules CD54, CD44, CD62L, CD49d on U937 was not significantly affected by the addition of both AD-MSCs-CM and HNDFs-CM to the basal control medium. After stimulation of U937 with TNFα (25 ng/ml), expression of CD54 (Figure 4A), CD44 (Figure 4B) and CD62L (Figure 4C) was increased and the addition of AD-MSCs-CM (at 1:1 dilution) was able to antagonize the increment (Figure 4A, B and 4C). In contrast, the addition of HNDFs-CM did not affect adhesion molecules increment induced by TNFα; only CD54 expression was slightly reduced (Figure 4A). Neither AD-MSCs-CM nor HNDFs-CM had any affect on CD49d expression (Figure 4D).

**Figure 5:**
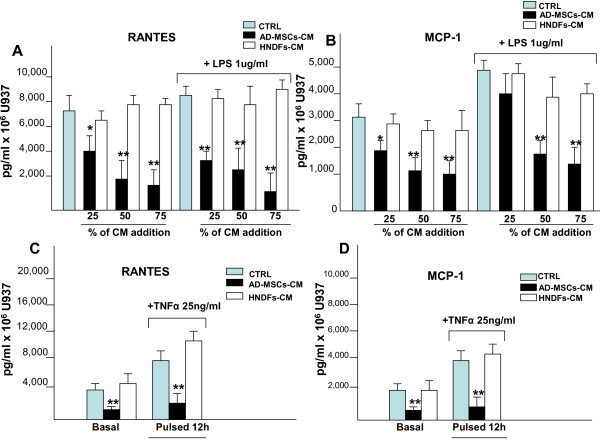
**Addition of AD-MSCs-CM, but not HNDFs-CM, to U937 blocked production of RANTES and MCP-by U937**. ELISA-kits were used to quantify the production of RANTES and MCP-1 by U937 under basal culture conditions, in the presence of inflammatory stimuli LPS(1 μg/ml) **(A and B) **and TNFα (25 ng/ml) **(C and D) **and in the presence of AD-MSCs-CM and HNDFs-CM. In **(A) **RANTES and in **(B) **MCP-1 production are blocked in a dose dependent manner by the addition of AD-MSCs-CM but not by HNDFs-CM under basal or in the presence of LPS. Similarly, in **(C) **RANTES and in **(D) **MCP-1 release is inhibited by the addition of AD-MSCs-CM to U937 stimulated or not with TNFα for 12 hrs. The columns in the figure are mean ± SD of three independent experiments run in triplicate. *p < 0.05 and **p < 0.01 versus CTRL.

**Figure 6:**
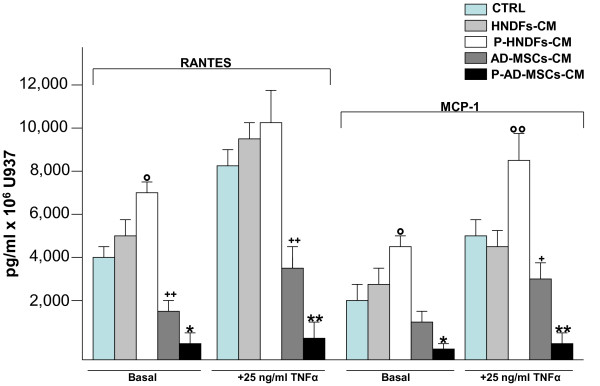
**Priming of AD-MSCs and HNDFs with TNFα induce an opposite effect on RANTES and MCP-1 release by U937**. AD-MSCs and HNDFs were primed for 12 hrs with TNFα 25 ng/ml. Thereafter cells were washed and further incubated for 24 hrs. At the end of incubation, CMs were collected from primed AD-MSCs (P-AD-MSCs-CM) and primed HNDFs (P-HNDFs-CM) and tested on U937 chemokines production. Note that, while the addition of P-AD-MSCs-CM to U937 completely blocked RANTES and MCP-1 release, in an opposite manner, P-HNDFs-CM improved their release. The columns in the figure are mean ± SD of two independent experiments run in triplicate. ° p < 0.05 and °° p < 0.01 versus HNDF-CM, *p < 0.05 and **p < 0.01 versus AD-MSCs-CM, + p < 0.05 and ++ p < 0.01 versus CTRL.

We also investigated the effect of AD-MSCs-CM and HNDFs-CM on CD11a and CD11b expression, adhesion molecules that are involved in inflammatory cells migration [25]. Only AD-MSCs-CM, but not HNDFs-CM, induced a slight decrement of CD11a and Cd11b expression on U937 (Additional file 6: Figure S6). We next investigated the capacity of AD-MSCs-CM and HNDFs-CM to affect the release of RANTES and MCP-1 inflammatory chemokines by U937, under basal culture conditions or in the presence of TNFα and LPS stimuli. AD-MSCs and HNDFs, per se, released very little amounts of both RANTES and MCP-1 (< 20 pg/ml for each chemokine) (data not shown). However, as shown in Figure 5, the addition of AD-MSCs-CM to U937 culture greatly inhibited RANTES (Figure 5A) and MCP-1 (Figure 5B) release in a dose dependent manner either in the presence or absence of LPS (1 μg/ml) inflammatory stimulation. By contrast, however, the addition of HNDFs-CM at every dilution tested did not (Figure 5A and 5B). The capacity of AD-MSCs-CM to block RANTES and MCP-1 chemokines release was even more potent if U937 was stimulated with TNFα for 12 hours (Figure 5C and 5D). Finally, we asked whether exposing AD-MSCs and HNDFs to the inflammatory cytokine TNFα may induce changes in their activity on U937 chemokines release. To this end, AD-MSCs and HNDFs were primed for 12 hours with TNFα (25 ng/ml), thereafter the medium was replaced with fresh medium, and 24 hours later CM was recovered and tested on U937 chemokines production. As shown in Figure 6, TNFα primed-AD-MSCs-CM (P-AD-MSCs-CM) added to U937 enhanced inhibition of RANTES and MCP-1 release by U937, either in the presence or absence of TNFα stimuli. Thus, priming with TNFα improved anti-inflammatory activity of AD-MSCs on U937 chemokines release. Interestingly, the addition of TNFα primed-HNDFs-CM (P-HNDFs-CM) produced, by contrast, a significant increment of chemokines release by U937 (Figure 6), indicating that HNDFs, under stimulation with TNFa, behave in an opposite manner.

## Discussion

Among the different stem cell phenotypes, adult MSCs represent an extremely valid cell option to apply to regenerative therapies. Besides their capacity to differentiate into many different cell lineages, including neural cells [26], MSCs display immunosuppressive [27], anti-inflammatory [15,23], anti-apoptotic [28] and angiogenic activity [15,28]-all distinguishing features of cells with potential regenerative properties [29]. In addition, MSCs can be isolated from many different adult human tissues [2-7]; in particular, BM and adipose tissue are sufficiently rich in MSCs content. For this reason, MSCs derived from BM and adipose tissue have been investigated more deeply and have been found to show similarities in terms of phenotypic profile, differentiation potential and *in vivo *regenerative capacity [7].

Procedures for their extraction and culture expansion have been fully described [2,3]. However, one of the major unsolved problems is the purity of MSCs preparation to use for therapeutic purposes. In fact, because of a lack of specific MSC markers, the presence of contaminating mature stromal cells, such as fibroblasts, cannot be distinguished and quantified, particularly in a fresh preparation or even in an expanded culture of AD-MSCs. Data from Lennon et al indicate that human BM-MSCs intentionally contaminated by dermal fibroblasts continue to elicit a positive osteochondrogenesis both *in vitro *and *in vivo *[30]. However, since there are very few comparative studies between AD-MSCs and fibroblasts and because the grade of purity of AD-MSC preparations may directly affect their *in vivo *efficacy [31,32], in the present study, we asked whether AD-MSCs are unique or whether they may share essential characteristics with fibroblasts. To this end, we compared the phenotypic and functional features of AD-MSCs and two dermal fibroblast cell lines. AD-MSCs were prepared from three different human donors following a standard procedure [15]. In contrast, we decided to use two commercial established fibroblast cell lines, to reduce the presence of cross-contaminating MSCs-like cells which could be present in fresh culture of dermal fibroblasts [10]. Moreover, commercial cell lines, are usually well characterized and composed of a more homogenous cell population if compared to fresh cell preparations. In fact, FC analysis of the two HNDF cell lines used confirmed the high level of stromal cell purity; cells were almost 100% positive for each mesenchymal marker tested (Table 1). In this regard, the analysis of the phenotypic profile confirms previous publications that indicate the absence of distinguishing markers in AD-MSCs and HNDFs [10,14]; they both expressed typical MSCs markers, were negative for endothelial and hematopoietic markers and even similarly negative for many stem cell markers, in particular Stro-1 and CD146, which are, by contrast, described on BM-MSCs [11,13]. Only ALDH activity, a stemness functional marker used for identification of stem cells and progenitors [16], was similarly present on both AD-MSCs and HNDFs. In this study, we also investigated the presence of immunogenic cell surface molecules on AD-MSCs and HNDFs. We found that CD54, CD80 and CD86 were similarly expressed, by contrast, CD40, a co-stimulatory protein found on APCs [20], was more highly expressed only on HNDFs. These data suggest that mature fibroblasts could have a higher immunogenic profile than AD-MSCs. In fact, the presence of very low levels of CD40 combined with the absence of CD14 and HLA-DR may not be sufficient to endow AD-MSC with APC function. In this study, we did not investigate the immunosopressive activity of AD-MSCs and HNDFs, because publications have already well described the immunosuppressive activity of both fibroblasts and MSCs [27,33].

We could not find any differences between AD-MSCs and HNDFs regarding morphological appearance in culture, growth rate and, more interestingly, in multipotential differentiation capacity. Hence our results are in contrast with Wagner et al [14] but support the finding of Lorenz et al who propose dermal fibroblasts for application in the therapy of wound healing because of their multilineage differentiation potential [10].

Besides this aspect, in our opinion this study has disclosed important new insights into functional differences between AD-MSCs and HNDFs. More specifically, it is known that HNDFs play an important role in angiogenesis, providing the extracellular matrix molecules necessary to support capillary morphognesis [34] and a balance of pro-angiogenic and anti-angiogenic factors to determine the final vascular density [35,36]. We did not investigate the production of extracellular matrix proteins produced by AD-MSCs and HNDFs in culture, but instead, we studied both the content of angiogenic growth factors, such as VEGFa, HGF, TGFβ1, Ang-1 and Ang-2 and PDGF released in the CM and, concomitantly, their activity on ECs proliferation and tube-like formations. We demonstrated, for the first time, that AD-MSCs were extremely more angiogenic then HNDFs. Indeed, if compared to HNDFs-CM, the level, in particular of VEGFa, HGF and Ang-1, was significantly higher in the AD-MSCs-CM as was its capacity to stimulate ECs proliferation. This data is consistent with our [15] and other previous reports [29] indicating the strong angiogenic potential of AD-MSCs and their potential use for the therapy of ischemic diseases [15,29]. Vice-versa, HNDFs appear less angiogenic. Although it is still under debate, if fibroblasts can be considered more mature cells than MSCs, our data may indicate that the angiogenic potential of MSCs could be inversely related with their grade of maturation, explaining, at least in part, the reason why dermal fibroblasts are less efficient than MSCs in promoting wound healing [37]. In addition AD-MSCs, but not HNDFs, were able to improve and stabilize HMECs tube-like formation, probably through their capacity to secrete significant quantities of Ang-1, an agiogenic factor involved in vascular morphogenesis [38]. This result expands on previous observations proposing that MSCs are different from fibroblasts and more similar to pericytes, [39] also because MSCs and pericytes are located in the wall of vasculature [6,40].

This study confirms the strong anti-inflammatory potential of AD-MSCs [15,23] and concomitantly highlights, for the first time, that HNDFs lack this property. In our opinion, this appears to be the most distinguishing feature between AD-MSCs and HNDFs. AD-MSCs were able to reduce adhesion molecules expression and inhibit the U937 release of the inflammatory chemokines RANTES and MCP-1, under different inflammatory stimuli. HNDFs did not affect any of the inflammatory activities of U937 monocytes. More interestingly, our results disclose a little-studied characteristic of AD-MSCs and HNDFs. Indeed, we observed that, under inflammatory stimuli, AD-MSCs and HNDFs behave in an opposite manner on U937 chemokines release. Upon priming with TNFα, AD-MSCs increased their capacity to block the release of RANTES and MCP-1, while HNDFs, by contrast, acquired pro-inflammatory activity, enhancing chemokines release by U937. To our knowledge, this is a new finding, that could be used not only to clearly distinguish between AD-MSCs and fibroblasts preparations, but also to improve MSCs regenerative properties when applied to cell therapy of ischemic diseases [41]. In addition, enhancing anti-inflammatory activity of MSCs may provide a better strategy to affect diseases where inflammation plays an important role in supporting progression.

## Conclusions

This study shows evidence that AD-MSCs and HNDFs share a number of similar phenotypic functional features, including the capacity to differentiate into different cell lineages. We demonstrate that, to distinguish AD-MSCs and HDNFs cultures, it is necessary to evaluate their angiogenic and, overall, their anti-inflammatory potential. AD-MSCs are significantly more angiogenic and anti-inflammatory than HNDFs. In addition, under stimulation with TNFα, AD-MSCs and HNDFs behave in an opposite manner: AD-MSCs improve anti-inflammatory activity, while HNDFs enhance inflammation. All together these data suggest that, before application in cell-based therapy, preparations of MSCs require testing for their angiogenic and anti-inflammatory capacity. Priming them with inflammatory cytokines may be useful for improving their therapeutic efficacy.

## List of abbreviations used

**AD-MSCs**: Adipose-derived MSCs; **AD-MSCs-CM**: conditioned medium-derived AD-MSCs; **ALDH**: aldehyde dehydrogenase; **Ang-1**: Angiopoietin-1; **Ang-2**: Angiopoietin-2; **AP**: Alkaline phosphatase; **APCs**: antigen presenting cells; **BM**: bone marrow; **BM-MSC s**: bone marrow derived MSCs; **CM**: conditioned medium; **Cx43**: Connexin 43; **ECs**: endothelial cells; FC: Flow cytometry; **HGF**: Hepatocyte growth factor; **HNDFs**: Human normal dermal fibroblasts; **HNDFs-CM**: conditioned medium-derived HNDF; **HMECs**: human microvascular endothelial cells; **HUVECs**: human umbilical vein endothelial cells; **MSCs**: mesenchymal stem cells; **MyoA**: myocardial actin; **LPS**: lipopolisaccaride; **P-AD-MSCs-CM**: priming-derived AD-MSCs-CM; **PDGF**: platelet derived growth factor; **P-HNDFs-CM**: priming-derived HNDFs-CM; **SMCs**: smooth muscle cells; **TGFβ1**: transforming growth factor β1; **TNFα**: tumor necrosis factor α; **TNP-C**: cardiac troponin; **VEGF**: vascular endothelial growth factor; **vWf**: von Willebrand factor

## Competing interests

The authors declare that they have no competing interests.

## Authors' contributions

All authors have read and approved the final manuscript. AB, CM designed experiments, differentiation experiments, performed *in vitro *experiments on U937, performed FACS analysis and ELISA assay. LB, MS Isolation and culture AD-MSCs, immunofluorescent characterization. SEN, LC, SC, and GI Performed experiments on ECs and Matrigel assay, isolation and culture of HUVECs and HMECs, AS, EAP supervised the manuscript, GA conceived, directed the study, and wrote the manuscript.

## Supplementary Material

Additional file 1 **Figure S1 Morphological appearance of AD-MSCs and HNDFs in culture**. The Figure shows the culture of AD-MSCs and HNDFs at early (P3) and at late (P10) *in vitro *passages. Note that AD-MSCs as well as HNDFs have a similar fibrablastic-like morphology (magnification 10×) Click here for file

Additional file 2 **Figure S2 AD-MSCs and HNDFs show a similar ALDH expression**. Aldefluor test was used to identify stem and progenitor cells with low side scatter that expressed high levels of ALDH. The Figure shows FC analysis of ALDH expression on both AD-MSCs and HNDFs. Note that both kinds of cell culture contained a similar percentage of ALDH positive cells. Click here for file

Additional file 3 **Figure S3 Marker's expression of AD-MSCs and HNDFs**. Note the high expression of mesenchymal markers CD90, CD44, CD105, CD73 and CD166 on both AD-MSCs and HNDF, whereas HNDFs expressed higher levels of CD40 and CD49d. Click here for file

Additional file 4 **Figure S4 AD-MSCs and HNDFs produce high level of Adiponectin after adipogenic differentiation**. Adiponectin expression was used to confirm adipogenic differentiation of AD-MSCs and HNDFs. The figure shows the FC of Adiponectin expression before and after adipogenic differentiation of AD-MSCs and HNDFs. Note that both kinds of cell culture expressed high level of Adiponectin after differentiation. Click here for file

Additional file 5 **Figure S5 AD-MSCs, and HNDFs, produce high levels of IL6 and IL8**. ELISA-tests were performed to detect IL6 and IL8 cytokines released by AD-MSCs and HNDFs in the CM. Note that both AD-MSCs, and HNDFs, release a high quantity of IL6 and IL8. Data are expressed as mean ± SD of the secreted factor per 10^6 ^cells after 72 hrs of incubation. Tests were run in triplicate and repeated twice. The background values contained in EGM control medium were subtracted Click here for file

Additional file 6 **Figure S6 AD-MSCs-CM, but not HNDFs-CM, reduced expression of CD11a and CD11b on U937 monocytes**. Note that the addition of AD-MSCs-CM (1:1), but not HNDFS-CM, to U937culture medium slightly reduced MFI of CD11a CD11b, particularly upon stimulation with TNFα. Click here for file
