# Supplementary figures and images for: Dermal fibroblasts display similar phenotypic and differentiation capacity to fat-derived mesenchymal stem cells, but differ in anti-inflammatory and angiogenic potential
Source: Vasc Cell. 2011 Feb 8;3:5. doi: 10.1186/2045-824X-3-5 (PMC3044104; doi:10.1186/2045-824X-3-5)

**AD-MSCs**

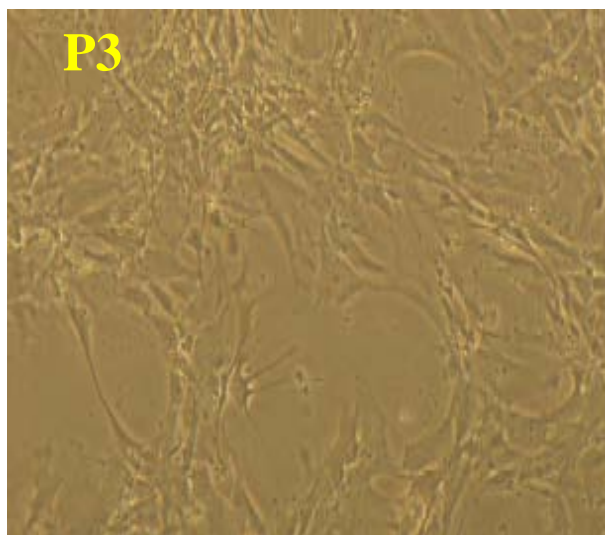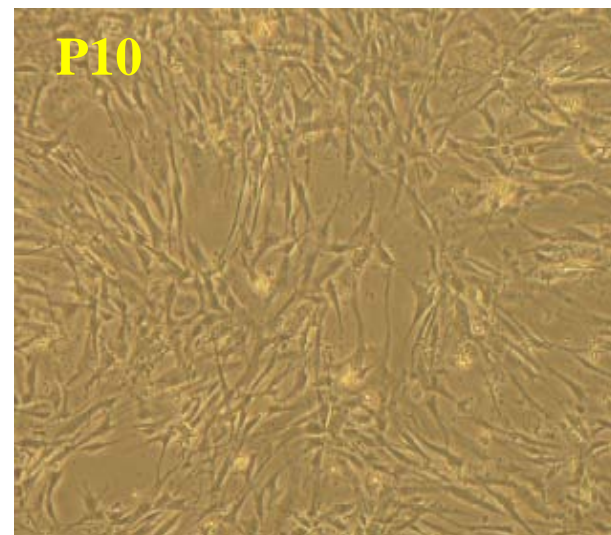

**HNDFs**

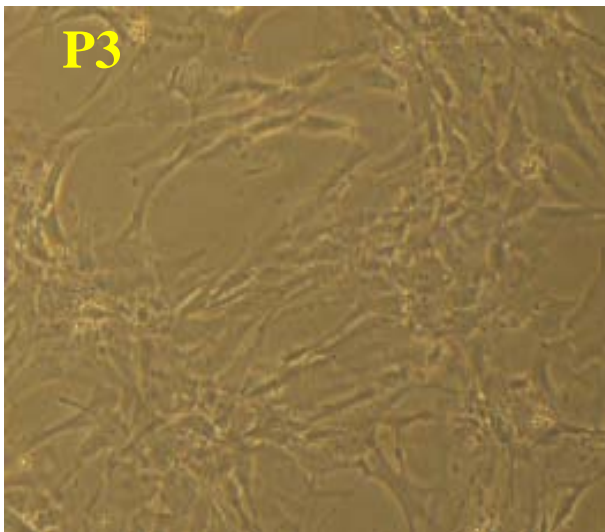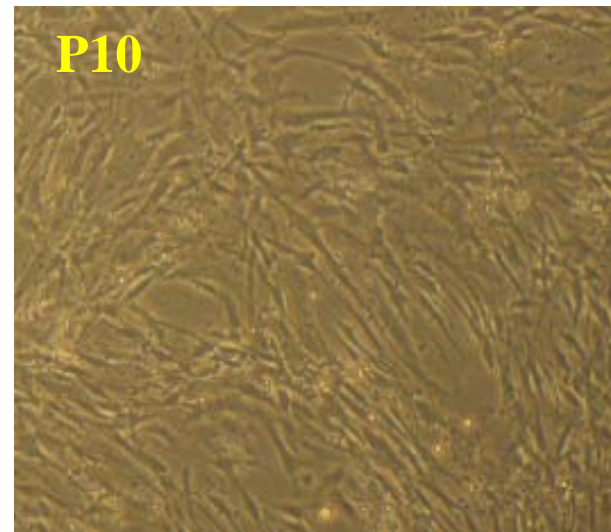

Supplement: Additional file 1 — Figure S1 Morphological appearance of AD-MSCs and HNDFs in culture. The Figure shows the culture of AD-MSCs and HNDFs at early (P3) and at late (P10) in vitro passages. Note that AD-MSCs as well as HNDFs have a similar fibrablastic-like morphology (magnification 10×) [file 2045-824X-3-5-S1.PDF]

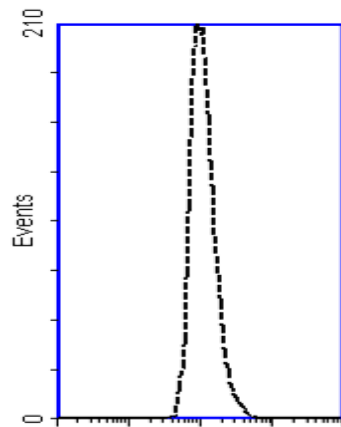

CTRL

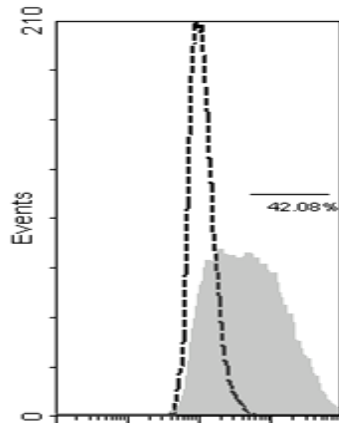

ALDH

■ M1: 42.08%

**AD-MSCs**

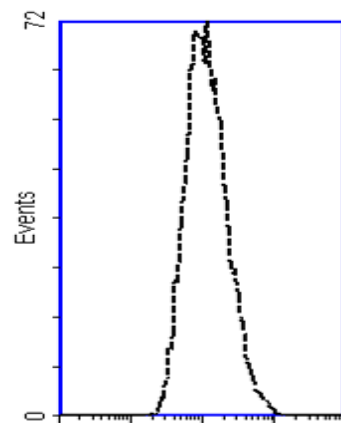

CTRL

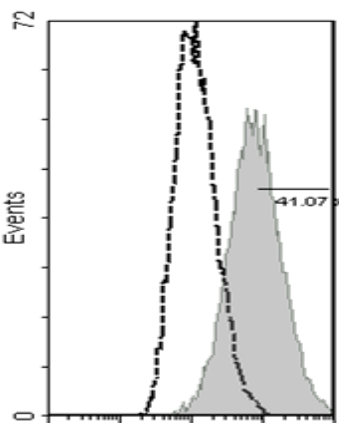

ALDH

■ M1: 41.07%

**HNDFs**

Supplement: Additional file 2 — Figure S2 AD-MSCs and HNDFs show a similar ALDH expression. Aldefluor test was used to identify stem and progenitor cells with low side scatter that expressed high levels of ALDH. The Figure shows FC analysis of ALDH expression on both AD-MSCs and HNDFs. Note that both kinds of cell culture contained a similar percentage of ALDH positive cells. [file 2045-824X-3-5-S2.PDF]

## HNDFs

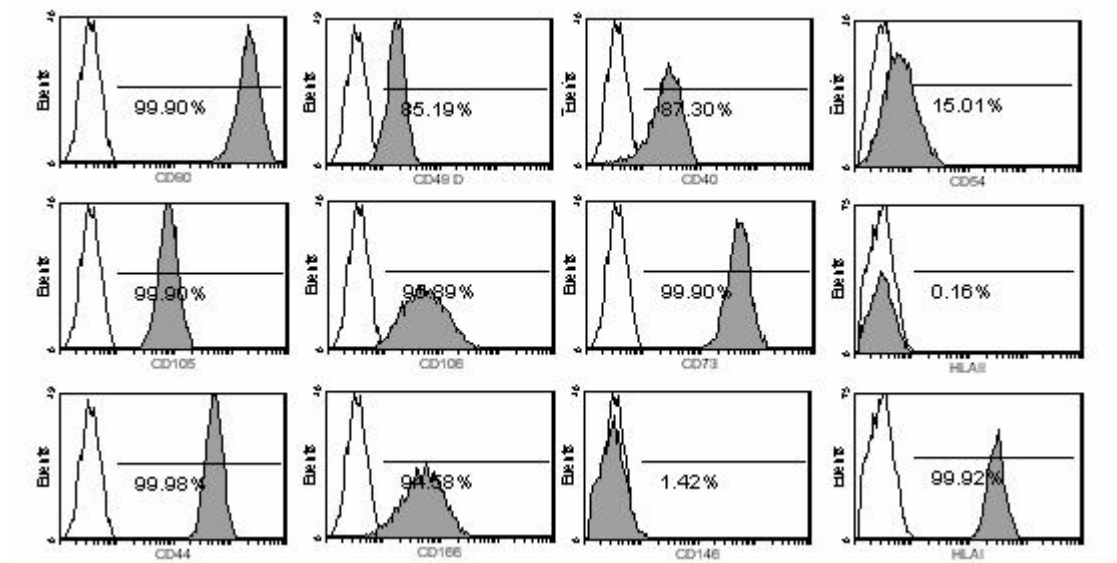

## AD-MSCs

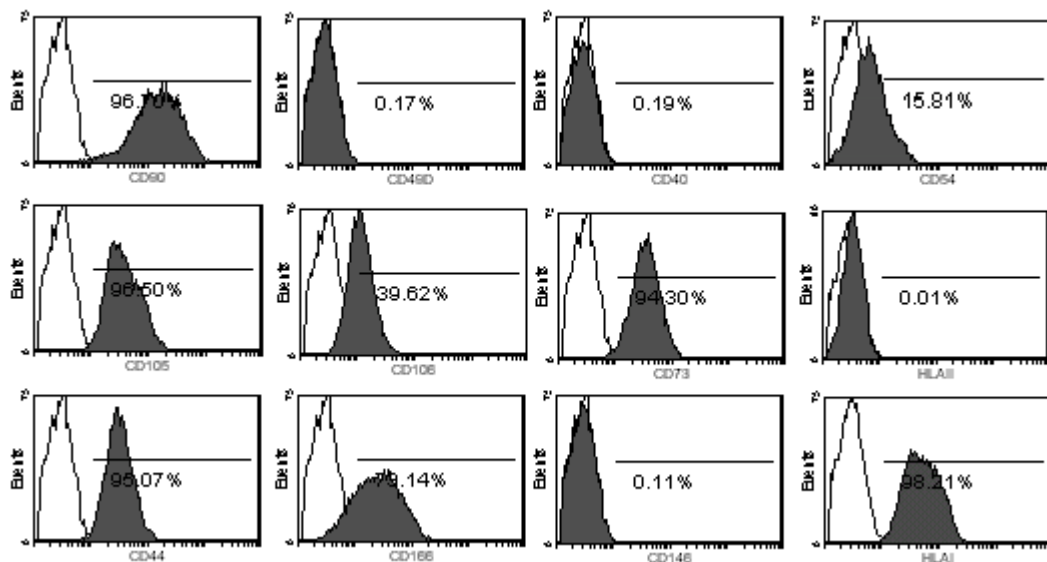

Supplement: Additional file 3 — Figure S3 Marker's expression of AD-MSCs and HNDFs. Note the high expression of mesenchymal markers CD90, CD44, CD105, CD73 and CD166 on both AD-MSCs and HNDF, whereas HNDFs expressed higher levels of CD40 and CD49d. [file 2045-824X-3-5-S3.PDF]

## AD-MSCs

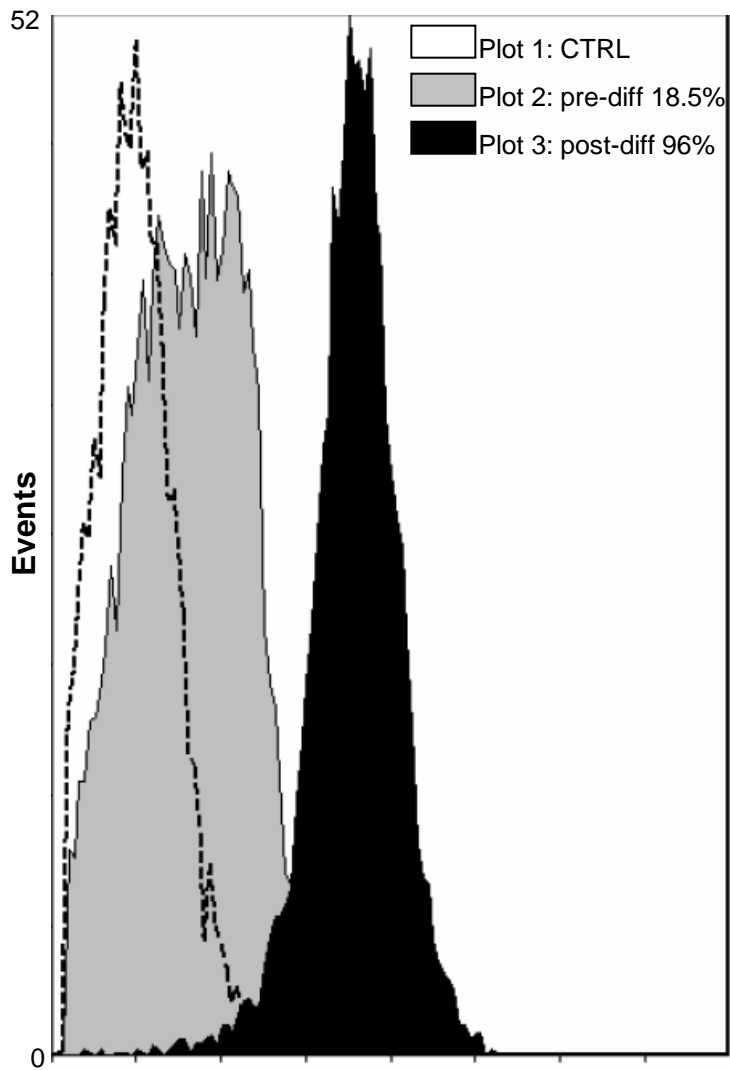

## HNDFs

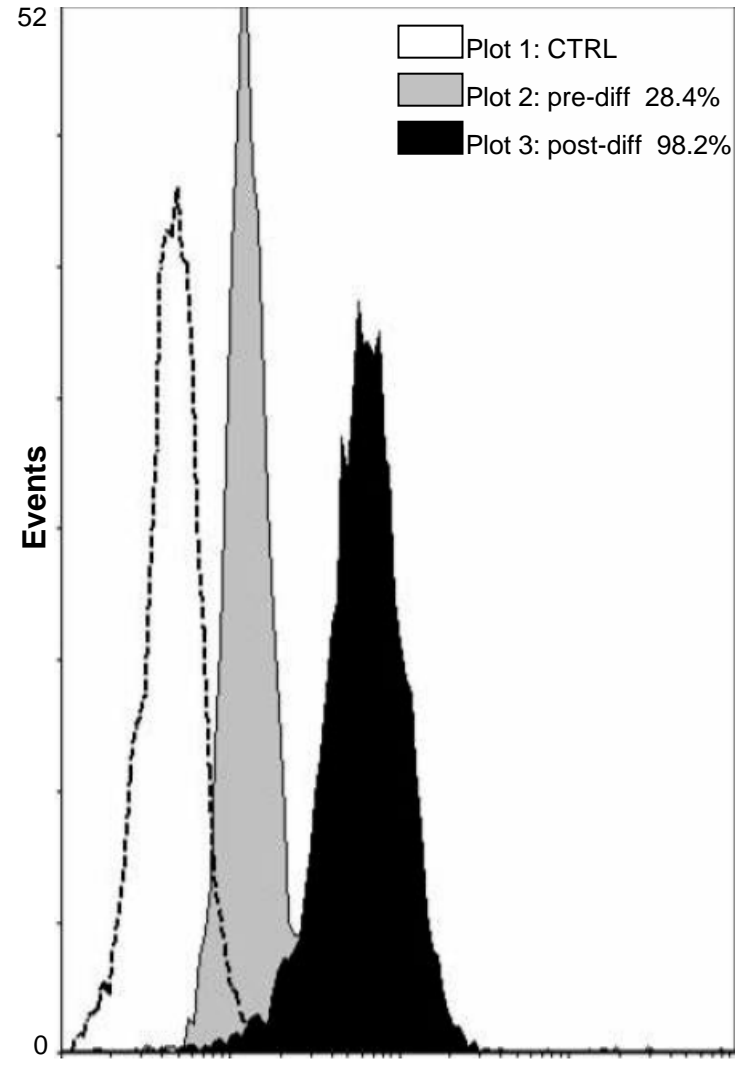

Supplement: Additional file 4 — Figure S4 AD-MSCs and HNDFs produce high level of Adiponectin after adipogenic differentiation. Adiponectin expression was used to confirm adipogenic differentiation of AD-MSCs and HNDFs. The figure shows the FC of Adiponectin expression before and after adipogenic differentiation of AD-MSCs and HNDFs. Note that both kinds of cell culture expressed high level of Adiponectin after differentiation. [file 2045-824X-3-5-S4.PDF]

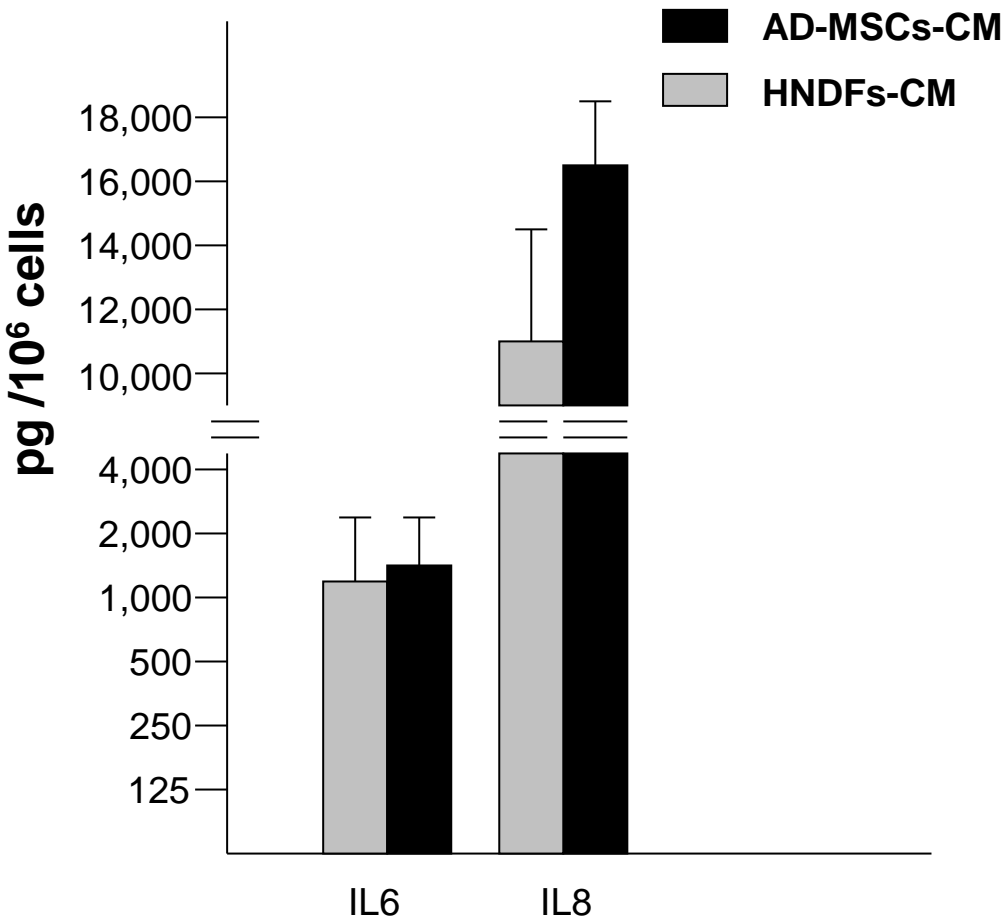

Supplement: Additional file 5 — Figure S5 AD-MSCs, and HNDFs, produce high levels of IL6 and IL8. ELISA-tests were performed to detect IL6 and IL8 cytokines released by AD-MSCs and HNDFs in the CM. Note that both AD-MSCs, and HNDFs, release a high quantity of IL6 and IL8. Data are expressed as mean ± SD of the secreted factor per 106 cells after 72 hrs of incubation. Tests were run in triplicate and repeated twice. The background values contained in EGM control medium were subtracted [file 2045-824X-3-5-S5.PDF]

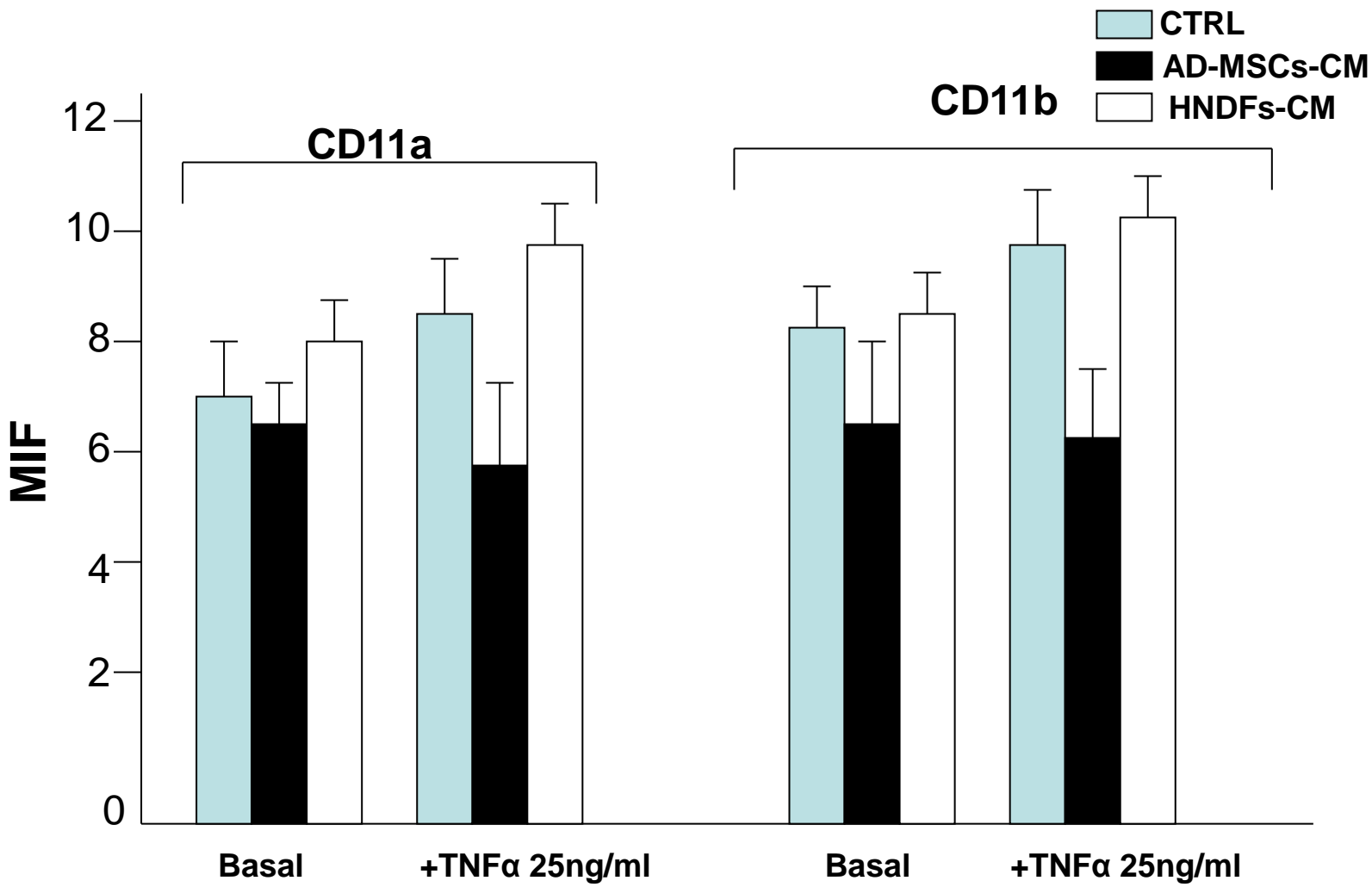

Supplement: Additional file 6 — Figure S6 AD-MSCs-CM, but not HNDFs-CM, reduced expression of CD11a and CD11b on U937 monocytes. Note that the addition of AD-MSCs-CM (1:1), but not HNDFS-CM, to U937culture medium slightly reduced MFI of CD11a CD11b, particularly upon stimulation with TNFα. [file 2045-824X-3-5-S6.PDF]
